# Prevalence of Elbow Joint Arthritis and Enthesitis in Rheumatoid Arthritis

**DOI:** 10.3390/jcm9051590

**Published:** 2020-05-24

**Authors:** Valentin Sebastian Schäfer, Florian Recker, Diana Vossen, Isabelle Geffken, Eva Matuschek, Wolfgang Hartung

**Affiliations:** 1 Department of Rheumatology and Clinical Immunology, Clinic of Internal Medicine III, University Hospital Bonn, 53127 Bonn, Germany; 2 Department for Obstetrics and Gynecology, University Hospital Bonn, 53127 Bonn, Germany; florian.recker@ukbonn.de; 3 Department for Internal Medicine and Rheumatology, Rheinisches Rheumazentrum St. Elisabeth Hospital, 40668 Meerbusch, Germany; diana.vossen@rrz-meerbusch.de (D.V.); Isabelle.geffken@gmail.com (I.G.); 4 Department of Internal Medicine, Dreifaltigkeits Hospital, 50389 Wesseling, Germany; eva.m.matuschek@t-online.de; 5 Department of Rheumatology/Clinical Immunology, Asklepios Medical Center, 93077 Bad Abbach, Germany; w.hartung@asklepios.com

**Keywords:** rheumatoid arthritis, ultrasound, elbow, enthesitis, prevalence

## Abstract

Objectives: The prevalence of elbow joint arthritis in rheumatoid arthritis (RA) assessed by ultrasound has not yet been investigated. Methods: We investigated 102 patients with RA and 50 patients without rheumatological disease. Both elbow joints were examined by ultrasound for effusion, hypervascularization, and enthesitis. A clinical examination was performed, and Disease Activity Score in 28 joints (DAS28), and visual analog scale for pain (VASp) were recorded. Arthritis was defined as joint effusion (≥grade II) and synovial hyperperfusion. Results: The RA cohort versus the control group displayed a joint effusion in 54.9% vs. 6.9%, a hypervascularization in 6.8% vs. 0%. Arthritis was detected in 36 RA patients (35.29%) and no one in the control group. Four (3.8%) RA patients and one (1%) control displayed enthesitis. The RA cohort showed a significant correlation between movement restriction and joint effusion (*p*-value = 0.001) as well as DAS28 (*p*-value = 0.02) and between DAS28 and ultrasound detected arthritis (*p*-value = 0.022). In an overall analysis, a highly significant correlation of VASp with movement restriction (MR) (*p*-value ≤ 0.001), the presence of joint effusion (*p*-value ≤ 0.001), and the diagnosis of RA (*p*-value ≤ 0.001) were observed. Interrater analysis of ultrasound imaging showed good agreement with Cohen’s kappa of 0.896. Conclusion: The prevalence of elbow arthritis in RA seems to be high, with 35.29%. Movement restriction is a good indicator, but not in all RA patients (32 vs. 70 patients without MR) compared to the control group (5 vs. 45 patients without MR). Reported pain correlates with joint effusion and MR (*p*-value ≤ 0.001).

## 1. Introduction

Rheumatoid arthritis (RA) is a chronic, systemic inflammatory autoimmune disease affecting approximately 1% of the population worldwide. It affects the synovial membranes resulting in synovitis as a primary abnormality and subsequently leads to bone destruction such as erosions and cartilage damage [1,2,3]. Small joints, especially of the hands are frequently involved [4], and joint destruction results in severe deformity and disability, but also large joints are commonly affected, as erosive changes of the elbow joint are seen in about 32 % [5]. An observational database analysis comparing the clinical disease presentation of newly diagnosed RA patients in Mexico, South Africa, India, and the Netherlands found a prevalence of 29%, 23%, 7%, and 7% for each country, respectively. Early and accurate diagnosis and subsequent treatment are necessary in order to avoid structural damage, which will result if the disease is left untreated [6]. 

Musculoskeletal ultrasound (MSUS) is, nowadays, increasingly used in the diagnosis of RA [7,8]. Radiographs have traditionally been the mainstay for imaging in patients with RA; findings such as soft-tissue swelling, periarticular osteopenia, joint space loss, joint subluxation, and marginal erosions are all features that may be seen. In the past two decades, MSUS greatly evolved and is now able to provide high-resolution imaging in assessing a wide range of pathologic conditions affecting the joints, including the synovial membrane, joint effusion, hyperperfusion of the synovia, bony surface, entheses and supporting soft tissues and ligaments.

It has been proven to be reliable in experienced hands [9,10] and offers many advantages over magnetic resonance imaging (MRI), including time and cost-effectiveness, superior spatial resolution, dynamic examination, and the possibility of performing the examination in a comfortable position for the patient. Compared with clinical examination, it is a more sensitive method for detecting synovitis and tenosynovitis [11]. So far, mostly small joints such as the metacarpophalangeal (MCP-), proximal interphalangeal (PIP-), metatarsophalangeal (MTP-) joints, and wrists were studied in RA, being the most affected joints. In contrast, large joints have not been extensively studied, and elbow involvement has only been incorporated in two ultrasound scoring systems by Naredo et al. [12] and Hartung et al. [13].

This is the first study applying the scanning planes for the elbow of the SOLAR (Sonography of large joints in rheumatoid arthritis) score by Hartung et al. [13], also proposed by the German Society for Ultrasound in Medicine (DEGUM) in order to assess the prevalence of elbow arthritis and enthesitis in patients with RA versus control patients without a rheumatic disease.

## 2. Patients & Methods

This national study started in January 2018 by founding a group of two board-certified rheumatologists with longstanding experience in MSUS (together > 15 years). A total of 152 patients were enrolled (102 patients with rheumatoid arthritis (67% female); 50 without the rheumatological disease (41% female)). The patients were recruited in the Department of Rheumatology and Clinical Immunology at the University Hospital Bonn, Germany, as well as at the Rhinish Rheumatology Center Meerbusch (Rheinisches Rheumazentrum Meerbusch), Germany, due to clinical cooperation. 

In addition to the MSUS examination, a clinical examination of the elbow for the detection of the degree of motion was performed, and the Disease Activity Score in 28 joints (DAS28) calculated. Furthermore, the visual analog scale for pain (VASp) and baseline characteristics (Table 1) were recorded. Patients who were diagnosed with RA had to meet the (American College of Rheumatology/European League Against Rheumatism) ACR/EULAR 2010 classification criteria [14]. The control group consists of patients in whom, after examination by a rheumatologist, a rheumatological disease was excluded. These patients mainly suffered from degenerative diseases and pain disorders. None of the patients or control patients in this study had received any surgical intervention of any side of the elbow joint.

### 2.1. Clinical Assessment

Both elbow joints of RA and control patients were clinically assessed for swelling, tenderness, and range of motion. The patient-rated visual analog scale for disease activity (range 0–100) was also reported. The Disease Activity Score in 28 joints (DAS28) was used to assess overall disease activity. Movement restriction (MR) was defined as a significantly decreased range of motion of the elbow joint of more than 10 percent at physical examination.

### 2.2. Ultrasound Examination

**Figure 1:**
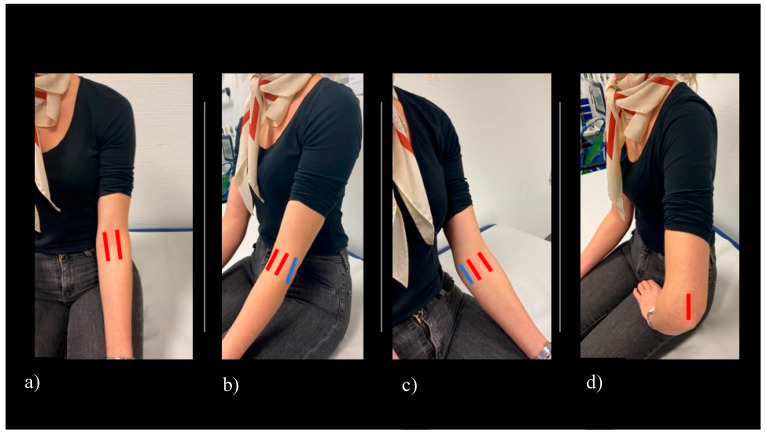
Scanning planes of the elbow applied in this study. (**a**) Humeroulnar and humeroradial longitudinal sections (displayed in red color), (**b**) longitudinal section over the lateral epicondyle (displayed in blue color), (**c**) longitudinal section over the medial epicondyle (displayed in blue color), (**d**) posterior longitudinal section (displayed in red color).

Ultrasound was performed using GE (General Electric) Logic S8 and e9 ultrasound machines, equipped with a multifrequency linear array probe (6–15 MHz). B-mode and power-Doppler (PD) settings for each US machine were optimized for image resolution and sensitivity by an application specialist and expert sonographer (DEGUM/European Federation of Societies for Ultrasound in Medicine and Biology (EFSUMB) level II). The following settings were applied for the examination of the elbow joint, B-mode frequency 12 MHz, image depth 4 cm, one focus point at 2.5 cm below the skin surface, Power Doppler frequency 10 MHz, and pulse repetition frequency 0.8 KHz. Sonographers were advised not to change these predefined settings, except for adjusting image depth and focus point position, if necessary. The elbow joint is examined while the patient is seated with his/her arm resting on the examination table [15]. Sonographers were allowed to move the elbow joint in order to find the best image position (Figure 1).

### 2.3. Scoring System

**Figure 2:**
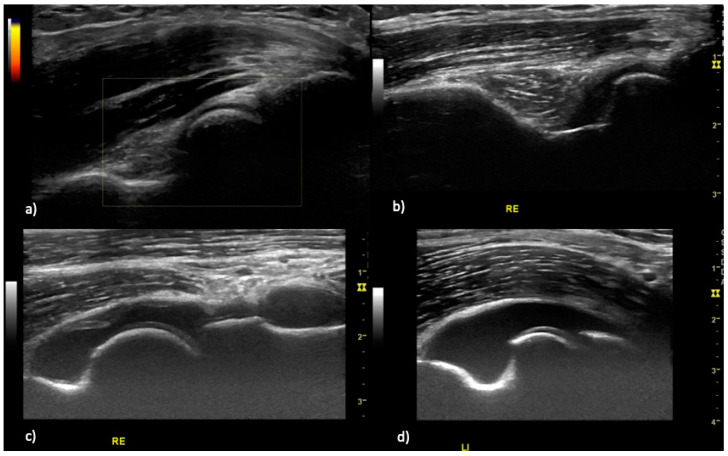
Joint effusion grading in rheumatoid arthritis patients. (**a**) elbow joint without effusion, (**b**) elbow joint effusion grade I with joint capsule distension parallel to capitulum or trochlea humeri, (**c**) elbow joint effusion grade II with straight joint capsule distension, (**d**) elbow joint effusion grade III with convex joint capsule distension.

**Figure 3:**
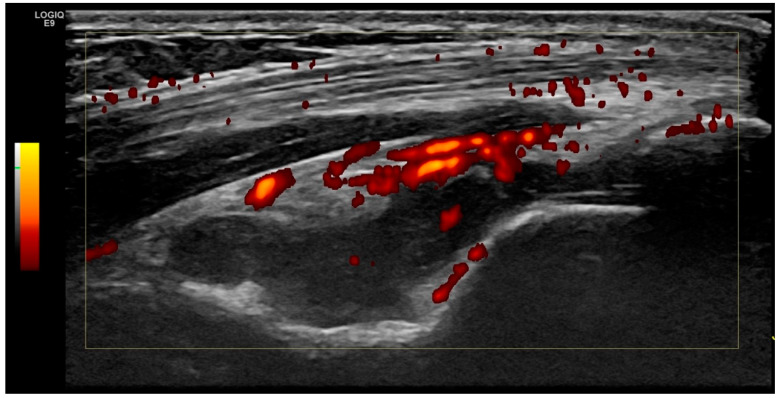
Elbow joint synovial hyperperfusion. Posterior longitudinal view of the left elbow joint with effusion grad II in the radial fossa and severe hyperperfusion of synovia.

Scanning and image acquisition were undertaken in line with the German Society for Ultrasound in Medicine (DEGUM) and the European Federation of Societies for Ultrasound in Medicine and Biology (EFSUMB) [16]. Planes for assessment of the elbow for effusion and hyperperfusion were extrapolated from the SOLAR score [13]. The predefined scanning planes (anterior longitudinal humeroradial- and humeroulnar, as well as posterior longitudinal scans) (Figure 1) were scored by B-mode and PD mode for both joint effusion (grades 0–3, Figure 2) and hyperperfusion (grades 0–1, Figure 3). Medial and lateral humeroradial und humeroulnar longitudinal scans were performed in order to assess for enthesitis as defined by the OMERACT (Outcome Measures in Rheumatology) Ultrasound group, subtask force for enthesitis [17].

### 2.4. Definition of Elbow Joint Arthritis and Enthesitis

Elbow joint arthritis was defined as joint effusion grade II (Figure 3) and intracapsular hyperperfusion (Figure 2). Enthesitis was defined as published by the OMERACT ultrasound group [17], as hypoechoic and thickened insertion of the tendon close to the bone (within 2 mm from the bony cortex) which exhibits Doppler signal if active, and which may show erosions and enthesophytes/calcifications as a sign of structural damage.

### 2.5. Inter- and Intra-Reader Reliability

Three readers took part in the US reliability sub-study. The reading of a specialist in MSUS (WH, DEGUM/EFSUMB level III) was used as the gold standard. Forty stored images were scored for effusion in gray-scale ultrasound (GSUS) and hyperperfusion in power Doppler ultrasound (PDUS) using blinded conditions. Analysis was performed semiquantitatively for effusion (grades 0–3) and binary for hyperperfusion (y/*n*) and enthesitis (y/*n*). For inter-reader testing, in total, 40 US images were rated three times. For intra-reader testing, the same 40 mixed images were scored after two months. Percentage of observed agreement (i.e., the percentage of observations that obtained the same score) was calculated. Inter- and intra-reader reliability of scoring static images was assessed according to kappa (κ) and Kendall’s tau statistics. The calculation of the inter-reader coefficient was exclusively based on the first measure of those pairs. Percentage of observed agreement (i.e., the percentage of observations that obtained the same score) was also calculated. Inter-reader reliability was studied by calculating the mean k for all pairs (i.e., the Light’s kappa) [18]. Inter-observer reliability was determined for each scanning plane separately. An agreement was computed for the elementary lesions of inflammation (i.e., the joint effusion, PD signal, and enthesitis). Intraobserver coefficients were evaluated on pairs of measures performed by the same sonographer for twenty images. Intra-item correlations with grey-scale images were determined using Kendall’s tau by using the distribution of responses and the potential need to collapse item response categories based on response frequencies [19]. Intra-item correlations concerning PD signal and enthesitits were determined according to kappa (κ). The images taken for the intra-reader agreement were rated again after one week by the same readers. 

### 2.6. Statistical Analysis

Statistical analysis was performed with SPSS statistical software, version 25.00 (IBM, Armonk, NY, USA). For quantitative parameters (e.g., the number of patients, age of the examined patients, and their disease activity), the mean standard deviation and range were determined for each. Significant changes were calculated by using *t*-test, x^2^-test and ANOVA calculation. *p* values less than 0.05 were considered statistically significant. Inter-reader and intra-reader agreement were calculated using kappa coefficients between the readers and kappa and Kendall tau coefficents [19] for the intra-reader agreement. The kappa coefficients were divided as follows: <0.0 = poor, 0–0.20 = slight, 0.21–0.40 = fair, 0.41–0.60 = moderate, 0.61–0.80 = substantial, and 0.81–1.0 = almost perfect agreement according to Landis and Koch [20].

### 2.7. Ethical Approval

Data acquisition and analysis were performed in compliance with protocols approved by the Ethical Committee of the University of Bonn (ethical approval number 345/18). Written informed consent was obtained from all participants prior to the study.

## 3. Results

### 3.1. Patient Characteristics

A total of 102 RA patients (67% female) with a mean age of 60 years (SD ± 13.8 years) and 50 control patients (82%female) with a mean age of 48 years (SD ± 16.7 years) were enrolled in the study. All RA patients fulfilled the 2010 ACR/EULAR classification criteria [14].

In the RA cohort, 33.3% (34) patients showed a relevant joint effusion (≥grade II) and 6.9% hypervascularization, whereas in the control group, no one showed a relevant joint effusion (≥grade II) and hypervascularization was not detected in any patients. The mean DAS28 in the RA cohort was 5.3 (±SD1.1) (Table 1). The mean VASp of RA patients was 6.41 (SD ± 2.3) and in the control group 2.42 (SD ± 2.9). In the RA cohort, there were four patients showing an enthesitis (3.9%) whereas in just one patient in the control group, an enthesitis was detected (Table 2).

**Figure 4:**
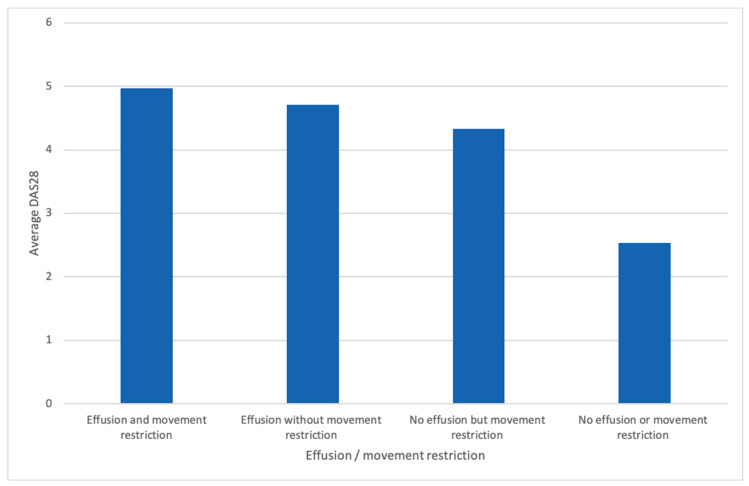
Correlation between the DAS28 and joint effusion/movement restriction. DAS28 = Disease Activity Score in 28 joints.

**Figure 5:**
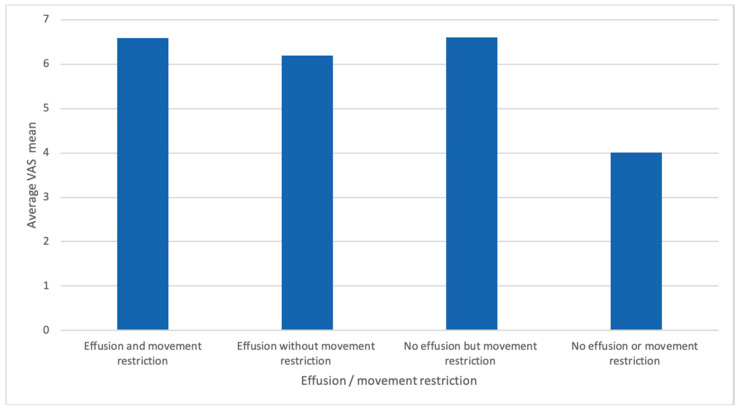
Correlation between VASp and effusion/movement restriction. VASp = visual analogue scale for pain.

In the RA cohort there was a significant correlation between movement restriction (MR) and joint effusion (*p*-value ≤ 0.001), as well as DAS28 (*p*-value = 0.02) and between DAS28 and joint effusion (*p*-value = 0.022). Further, we did not observe a significant difference between MR and single-elbow joint involvement in the RA cohort. The overall cohort (RA and control patients) showed a significant correlation of DAS28 with MR and joint effusion (Figure 4). In the total cohort, a correlation of VASp with MR (*p*-value 0.001) was detected. Further, a strong correlation between VASp with joint effusion (≥grad I) and MR (*p*-value ≤ 0.001) was found (Figure 5). In the RA cohort, 36 of 102 patients (35.29%) fulfilled our criteria of elbow joint arthritis (hyperperfusion and effusion > II) whereas in the control group, no one fulfilled these criteria (0/50). Interestingly, the arthritis was in only 15% of RA patients symmetrical, and in those patients also a decreased range of motion was symmetrically detected. Enthesitis was only observed unilaterally.

### 3.2. Intra- and Interreader Reliability

The intra-reader agreement using Kendall tau’s and kappa’s statistics showed the following results by both raters: rater 1 (Valentin Sebastian Schäfer, VSS) and rater 2 (Diana Vossen, DV) showed both a very high intra-reader agreement by scoring twenty images (Appendix A Table A1). For the semiquantitative scoring (0–3) of the stored images for joint effusion and hypervascularization in PDUS, as well as enthesitis, the overall kappa values for the inter-reader agreement were 0.824 (mean inter-reader agreement 87.5%) for joint effusion in GSUS and 0.694 (mean inter-reader agreement 93%) for hyperperfusion in PDUS. The following results for inter-reader reliability were observed: the mean kappa values for the semi-quantitative scoring for the joint effusion of the elbow were 0.824 with a significance level of 0.001 (Table 3).

## 4. Discussion

The purpose of our study was to determine the prevalence of elbow arthritis and enthesitis in rheumatoid arthritis and control patients by MSUS imaging and to correlate the findings to parameters of disease activity. To our knowledge, this is the first longitudinal MSUS study of the elbow joint in RA and controls. Using experienced musculoskeletal sonographers, we focused on the evaluation of joint effusion, synovial hypervascularization, and enthesitis by a defined and internationally applied ultrasound methodology, adhering to OMERACT protocol.

We found the prevalence of elbow joint effusion to be high with 54% (56/102) in the RA cohort vs. 16% (8/50) in the control group. Though effusion is unspecific and does not have to be a sign of arthritis, the prevalence found is certainly unexpectedly high. There is little evidence on the prevalence of elbow arthritis in RA [21]. Porter BB et al. [22] reported in 1974, an incidence of severe elbow joint disability by clinical examination in 225 RA patients of 25% in one or both upper limbs. Another publication from 1979 reported a prevalence of 50% of elbow arthritis [23]. Lehtinen et al. [24] described a radiographic prevalence of elbow joint arthritis of 61% in a cohort of seropositive and erosive rheumatoid arthritis patients. There are no data on ultrasound or MRI detected prevalence of elbow arthritis to date. Applying our ultrasound definition of arthritis, hyperperfusion and joint effusion > I we detected in 36/102 (35.29%) of RA patients and in none of the control patients an elbow arthritis. Though it seems that our applied ultrasound definition is applicable. Four RA patients (3.9%) and one (2%) control patient displayed an enthesitis, as defined by the OMERACT ultrasound definition of enthesitis. To date, there is growing evidence that enthesitis also exists, and RA and is not specific for psoriatic arthritis or spondyloarthritis, as previously thought [25,26,27,28]. Ebstein et al. [29] examined the prevalence of enthesitis seen on MUS in spondyloarthritis (*n* = 41), RA (*n* = 30) and healthy controls (*n* = 26). The authors found an enthesitis of the lateral epicondyle of the elbow in 29/82 (35%) of spondyloarthritis patients in 12/60 (20%) of RA patients as well as in 3/52 (5.8%) of controls. The medial epicondyle was not examined by authors, and the OMERACT enthesitis definition was not applied, as it had not yet been published, which surely explains the higher observed percentage, as the OMERACT ultrasound criteria for enthesitis are rather specific.

The presence of movement restriction does not seem to be a good indicator for elbow joint arthritis in RA, as it was only present in roughly a third—32/102 (31%) of patients with ultrasound had defined arthritis. Taking into account that this might be a rather low-grade chronic inflammation, as a sign of residual disease activity. However, if the elbow joint was painful, commonly MR and joint effusion were found (*p*-value 0.001), as well as a higher DAS28 score (*p*-value 0.02). The overall collective showed a significant correlation of DAS28 with MR and joint effusion, making this commonly applied marker of disease activity a valuable tool, which also seems to correlate well in the case of elbow arthritis. Obviously, pain seems to be an important parameter, as it is highly significant correlated with MR (*p*-value 0.001), the presence of joint effusion (*p*-value 0.001) and the diagnosis of RA (*p*-value 0.001).

Micu et al. [30] examined the impact of MSUS on clinical diagnosis and different treatment regimens. The authors described that MSUS of the elbow joint led in 80% to a change of final diagnosis and proved to be very helpful, while in all other anatomic areas examined, the proportion was lower.

Our findings support the routine ultrasound assessment of the elbow joint by MSUS in RA, as MR is only present in about one third of patients, for example by applying the SOLAR score [13], which has previously proven to be applicable in daily clinical practice. Enthesitis of the elbow joint seems to be rare but is present in a low proportion of patients. 

Our study has limitations. The control group was younger, with an average of 12 years of age, which could have affected the results, since with increasing age, inflammation may also be observed in healthy controls on MRI [31]. Further, data on disease duration and therapy were not available, as we relied on the DAS28 and VASp for disease activity. The observed excellent reliability may depend on the severity of the pathologic findings, as this was a longitudinal study, we did not select patients according to their disease severity. Although similar US equipment was used, even machines of the same type may exhibit different image features. Our study was performed with current high-quality modern 6–18 MHz probes. Further, only the general pain of the patients was recorded and not the specific pain during the physical examination. Another limitation is that we did not compare MSUS assessment with MRI examination, although the correlation of MSUS with MRI has been proven [9,10,32,33].

MSUS may detect elbow joint pathology in a significant number of patients, especially when a rapid and efficient medical service is desirable for optimizing health resources in an outpatient setting. The strength of the current study is the sample size and that the analyses included both RA and control patients, making the findings relevant to clinical practice. In addition, we used a predefined scoring system, the SOLAR score [13], for the evaluation of the elbow joint. In the current data-sets, we found bilateral affection applied to our arthritis definition in 15.68 percent (16/102). In such cases, we have no recommendation regarding which side to choose for longitudinal follow-up studies, and the ultrasonographer may freely choose which side to scan.

## 5. Conclusions

The prevalence of ultrasound detected elbow joint arthritis in rheumatoid arthritis is high, with 35.29%. Reported elbow pain correlates well with joint effusion and movement restrictions in elbow joint arthritis. It seems that ultrasound assessment of the painful elbow joint in rheumatoid arthritis might be helpful, even if no movement restriction is found on clinical examination.

## Figures and Tables

**Table 1:** Patient characteristics.

	Rheumatoid Arthritis Group	Control Group
Age	60 (±SD 13)	48 (±SD 16)
Gender male/female	34 (33 %)/68 (67%)	9 (18%)/41 (82%)
DAS 28	3.57	n.a.
high activity (>5.1)	high activity: 58 (56.90%)	
moderate activity (3.2–5.1)	moderate: 42 (41.20%)	
low activity (2.6–3.2)	low: 1 (1.00%)	
remission (<2.6)	clinical remission: 1 (1.00%)	
VAS pain (0–10)	6.41 (SD 2.30)	2.42 (SD 2.90)

DAS 28: Disease Activity Score-28; VAS: Visual analogue scala; SD: standard deviation.

**Table 2:** Clinical examination and ultrasound results.

	RA Group			Control Group		
	Right Side	Left Side	Both Sides	Right Side	Left Side	Both Sides
Movement restriction	26 (25.50%)	22 (21.60%)	16 (15.68%)	3 (6.00%)	3 (6.00%)	1 (2.00 %)
Any Joint effusion	42 (41.20%)	40 (39.20)	26 (25.00%)	6 (12.00%)	4 (8.00%)	2 (4.00%)
Hypervascularisation	4 (3.90 %)	3 (2.90 %)	0 (0.00%)	0 (0.00%)	0 (0.00%)	0 (0.00%)
Ultrasound arthritis definition (Effusion ≥Grade 2 and/or hypervascularisation)	27 (26.47%) (grade > II: 25, hypervascularisation: 2)	25 (24.51) (grade >II: 24, hypervascularisation: 1)	16 (15.68%)	0 (0%)	0 (0.00%)	0 (0.00%)
Enthesitis	1 (1.00 %)	3 (2.00%)	0 (0.00%)	1 (2.00%)	0 (0.00%)	0 (0.00%)

**Table 3:** Inter-reader reliability and assessment in 40 patients with rheumatoid arthritis.

	Prevalence (Mean)	Observed Agreement	K (Kappa)	Standard Deviation
B-mode (0 to 3)	Grade 0: 42% (17/40)	DV/VSS: 92.5% (37/40) DV/WH: 85% (34/40) VSS/WH: 85% (34/40)	DV/VSS: 0.896 DV/WH: 0.791 VSS/WH: 0.787	DV/VSS: 0.058 DV/WH: 0.077 VSS/WH: 0.079
	Grade I: 20% (8/40)			
	Grade II: 18% (7/40)			
	Grade III: 20% (8/40)			
PD-mode (y/*n*)	Grade 0: 87.5% (35/40)	DV/VSS: 95% (38/40) DV/WH: 95% (38/40) VSS/WH: 90% (36/40)	DV/VSS: 0.724 DV/WH: 0.805 VSS/WH: 0.553	DV/VSS: 0.183 DV/WH: 0.132 VSS/WH: 0.190
	Grade I: 12.5% (5/40)			

PD: power Doppler, DV: Diana Vossen, VSS: Valentin Sebastian Schäfer, WH: Wolfgang Hartung.

## Abbreviations

DEGUM	Deutsche Gesellschaft für Ultraschall in der Medizin (German Society for Ultrasound in Medicine)
DV	Diana Vossen
EFSUMB	European Federation of Societies for Ultrasound and Biology
MSUS	Musculoskeletal ultrasound
VSS	Valentin Sebastian Schäfer
WH	Wolfgang Hartung

## Appendix A

**Table A1:** Intra-reader reliability and assessment in 20 patients with rheumatoid arthritis.

	Rater 1: VSS		Rater 2: DV	
	Observed Agreement	Significance	Observed Agreement	Significance
Kendall tau (GSUS)	0.787	0.000	0.813	0.000
Kappa value (PDUS)	1.000	0.000	0.459	0.015

VSS: Valentin Sebastian Schäfer, DV: Diana Vossen. GSUS: gray scale ultrasound, PDUS: power Doppler ultrasound.
